# Risk of bleeding in surgical patients treated with topical bovine thrombin sealants: a review of the literature

**DOI:** 10.1186/1754-9493-2-5

**Published:** 2008-03-18

**Authors:** Matthew W Reynolds, John Clark, Sheila Crean, Srinath Samudrala

**Affiliations:** 1 Epidemiology and Database Services, United BioSource Corporation, Medford, MA, USA; 2 Epidemiology and Risk Managment, United BioSource Corporation, Medford, MA, USA; 3 Institute for Spinal Disorders, Cedars-Sinai Medical Center, Los Angeles, CA, USA

## Abstract

**Background:**

One of the most anticipated, but potentially serious complications during or after surgery are bleeding events. Among the many potential factors associated with bleeding complications in surgery, the use of bovine thrombin has been anecdotally identified as a possible cause of increased bleeding risk. Most of these reports of bleeding events in association with the use of topical bovine thrombin have been limited to case reports lacking clear cause and effect relationship determination. Recent studies have failed to establish significant differences in the rates of bleeding events between those treated with bovine thrombin and those treated with either human or recombinant thrombin.

**Methods:**

We conducted a search of MEDLINE for the most recent past 10 years (1997–2007) and identified all published studies that reported a study of surgical patients with a clear objective to examine the risk of bleeding events in surgical patients. We also specifically noted the reporting of any topical bovine thrombin used during surgical procedures. We aimed to examine whether there were any differences in the risk of bleeds in general surgical populations as compared to those studies that reported exposure to topical bovine thrombin.

**Results:**

We identified 21 clinical studies that addressed the risk of bleeding in surgery. Of these, 5 studies analyzed the use of bovine thrombin sealants in surgical patients. There were no standardized definitions for bleeding events employed across these studies. The rates of bleeds in the general surgery studies ranged from 0.1%–20.2%, with most studies reporting rates between 2.6%–4%. The rates of bleeding events ranged from 0.0%–13% in the bovine thrombin studies with most studies reporting between a 2%–3% rate.

**Conclusion:**

The risk of bleeds was not clearly different in those studies reporting use of bovine thrombin in all patients compared to the other surgical populations studied. A well-designed and well-controlled study is needed to accurately examine the bleeding risks in surgical patients treated and unexposed to topical bovine thrombin, and to evaluate the independent risk associated with topical bovine thrombin as well as other risk factors.

## Background

It is well accepted that there are both risks and tremendous health benefits associated with surgical procedures of all types. Surgical risks vary in severity and type and are driven by a complex mix of factors including surgical factors (e.g., type of surgery), surgeon characteristics (e.g., experience with conduction specific procedures), patient factors (age, gender, prior history of surgery, prior history of adverse events, comorbidities, etc.), other treatment factors (e.g., use of anticoagulants), and random chance. One of the most anticipated, but potentially serious complications in surgery is the risk of bleeding events. Among the many potential causes of bleeding complications in surgery, the use of bovine thrombin has been anecdotally identified as a possible cause of increased bleeding risk. Most of these reports of bleeding events in association with the use of topical bovine thrombin are case reports and small case series from which no clear cause and effect relationship could be established [1-16]. A more recent study failed to establish any significant differences in the rates of bleeding events between those treated with bovine thrombin and those treated with either human or recombinant thrombin [17].

Bovine thrombin has been used as a commercial hemostat for more than 60 years, and whether used alone or in combination with other clotting agents, it effectively helps to control surface bleeding in a variety of cardiovascular, vascular, neurological, orthopedic, transplant, and gynecologic surgeries [5,18,19]. Although bovine thrombin is often used as a hemostat in surgical procedures, only a few published studies have quantified the safety and efficacy of bovine thrombin in surgery. It is well documented in recent years that human exposure to bovine thrombin preparations has been associated with an allogentic anti-bovine response, as well as cross reactivity to human coagulation factors. High titers of antibodies directed against bovine clotting factors seem well documented as an immediate and common consequence of surgery and as a lifetime risk for those patients who receive multiple exposures of exogenous factors [9,20]. However, the clinical significance of this immunization on resulting adverse events is not clear or well documented. The adverse events of most interest, theoretically and clinically in surgery and treatment with bovine thrombin, are major bleeding events. If the antibody development associated with bovine thrombin exposure were to have an impact on the incidence of any adverse events, the most likely outcome would be the risk of major bleeds. Because the expected or background risk of bleeding events in surgery is not well documented, especially for surgery during which bovine thrombin is utilized, this literature review intends to examine and report the incidence of major bleeding events in published studies of surgery, as well as those specifically reporting bovine thrombin exposure and to determine whether the rates are notably different.

## Methods

We conducted a literature search of MEDLINE (via PubMed) for English language studies published within the past 10 years (1997–2007). We conducted our search of MEDLINE using a combination of text terms and Medical Subject Headings (MeSH) terms focused on surgery and bleeding events. We reviewed all the abstracts and retrieved all papers that met our eligibility criteria. We included all published studies that reported a study of surgical patients with a clear primary or secondary objective to examine and/or quantify the risk of bleeding events in that surgical population. For review articles that addressed this issue, we examined the references, retrieved all potentially eligible articles, and reviewed those as well.

Study inclusion and exclusion criteria are detailed below:

### Exclusion criteria

• Letters, editorials, news, case-reports, commentaries, and reviews

• Animal or in vitro studies

• Language other than English

• Study participants who were not undergoing surgery

• Studies of organ transplants

• Studies without a primary/secondary objective of examining or quantifying the risk of bleeding events in surgical patients

### Inclusion criteria

• Studies published from 1997–2007

• English language

• Studies of surgical patients with a clear primary or secondary objective of examining or quantifying the risk of bleeding events

Additionally, we conducted a secondary search using text and MeSH terms to identify surgical studies of bovine thrombin. We expected the yield of this search to be much smaller, so these studies were not required to have a primary/secondary objective of studying the risk of bleeding events, rather all studies that reported bleeding events were included. We also applied the same inclusion/exclusion criteria regarding publication date, English language, and organ transplant studies to the bovine thrombin studies.

We reviewed and summarized all studies regarding surgery type, sample size, geographic location, study design, definition of bleeding events, and risk of bleeding events.

## Incidence of bleeds in surgery studies

We identified 16 studies that met our inclusion criteria, which are presented in Additional file 1, Table 1 [21-36]. Seven of these studies were cardiovascular surgery studies [25,27,28,31,32,35,36], three were pancreatic surgery [21,24,29], and the others included major abdominal surgery [23], major orthopedic surgery [33], non-cardiac general surgery [34], laparoscopic cholecystectomy [26], prostatic resection [22], and laparoscopic and thoracoscopic surgery [30]. Seven of the studies included more than 1,000 patients[23,24,26,28,30,31,33]. Three studies were based in the United States, nine were based in Europe, and one each was based in Canada, Israel, Australia, and India.

Most of the studies had a very detailed definition of bleeding events, while several just reported specific events such as re-exploration for bleeding or delayed massive hemorrhage. There were no standardized definitions for bleeding events, major bleeds, etc., employed across these studies. The rates of bleeds ranged from 0.1% for fatal bleeds in orthopedic surgery [33] to 20.2% for bleeding complications in cancer patients undergoing pancreaticoduodenectomy [21]. The incidence of bleeding events in coronary artery bypass graft (CABG) patients was very similar across the six studies of CABG patients [27,28,31,32,35,36], ranging from 3.1% [28] to 5.4% [35], with the other studies reporting rates between 4%–4.6%. Three of the CABG studies reported rates of bleeding events to be between 4.0%–4.8% [27,31,32]. Those same CABG studies also showed an increased risk of bleeds with pre-operative aspirin use (7.8% risk of bleeds; [36]) and clopidogrel (8% risk with clopidogrel use within three days prior to surgery). The rates of bleeding events are presented in Additional file 1, Table 1 and show that most studies report a rate of 2.6%–4%.

The three pancreatic surgery studies showed a wide range of bleed rates from 2.3% for delayed massive hemorrhage [24] to 8.8% with at least one bleeding event [29], and 20.2% in the cancer patients [21]. The study reporting 8.8% risk of bleed events also reported a 47% fatality rate among those patients experiencing hemorrhage.

The prostatic resection study of 826 patients comparing two approaches found a 3.6% risk of delayed hemorrhage and a 4%–4.8% risk of total bleeds depending on the surgical approach [22]. A national study of laparoscopic cholecystectomy patients from Hungary reported a 1.44% risk of postoperative bleeds via a hospital survey of bleeding events [26]. A national registry of laparoscopic surgery patients from Switzerland also reported a lower rate of bleeding events with a 3.3% incidence of major bleeds [30]. A randomized trial of heparin thromboprophylaxis for major abdominal surgery in the United Kingdom found rates of total bleeds of approximately 11% in both the low molecular weight heparin and standard heparin groups with about an 8% incidence of major bleeds. The risk of bleeds in this study during the operation (5.8%) appeared to be similar to the rate of bleeds and post-operatively (6.5%) [23].

A study of non-cardiac general surgery patients in the Netherlands reported a 2.6% risk of all bleeds and a 0.9% risk of major bleeds [35]. A study of major orthopedic surgery in the United States reported an overall 2.6% risk of major bleeds and a 0.7% risk of re-operation for bleeding [33]. The study also noted that the rates of major bleeds varied by surgery type with 4.9% in total hip replacement, 1.3% in major knee surgery, and 2.8% in hip fracture repair. They also reported that factors such as older age, male gender, and presence of comorbid hepatic/renal disorders significantly increased the risk of a major bleed event.

None of these studies of surgical patients reported whether any or all of the patients in the study received treatment with any bovine thrombin-containing products.

## Incidence of bleeds in bovine thrombin surgery studies

Our review of the literature identified five studies that reported the risk of bleeds in a surgical population treated with bovine thrombin [5,9,17,37,38]. The studies ranged in size from 21 patients [37] to 411 patients [17], and are summarized in Additional file 1, Table 2. The rates of bleeding events ranged from 0.0% [37] to 13% (human thrombin treatment arm; [17], with most studies reporting between a 2%–3% rate of bleeding events. The rates are also displayed in Additional File 1, Table 2, showing that average rate of bleeds is in the range of 2%–3.5%, similar to what was seen in the other published surgical in Additional file 1, Table 1.

A study of 21 cardiothoracic surgical patients exposed to a fibrin glue of bovine fibrinogen and bovine thrombin during surgery were tested for anti-bovine fibrinogen titers prior to surgery and followed for 233 days [37]. All patients demonstrated elevated anti-bovine fibrinogen antibodies. When nine patients with the highest anti-bovine fibrinogen were retested, they displayed high titers for bovine thrombin and human thrombin as well. However, none of the 21 patients developed postoperative bleeding complications, including those with initially high fibrinogen titers. A second study was conducted by Dorion et al [5] that examined stored blood samples and hospital records and identified 120 patients previously exposed to bovine thrombin. Of these patients who could be identified from medical records, 102 had been exposed once to bovine thrombin and 18 had been exposed multiple times. The study also included 114 patients who had been unexposed to bovine thrombin. The study found that of the patients who had been exposed to bovine thrombin, 10% had antibodies to bovine thrombin; in patients singly exposed, the rate was 5%; and in patients exposed multiple times, the rate was 39%. The incidence of serious bleeds in bovine-thrombin exposed patients was 1.7% (two of 120 patients) of the total exposed patients while it was higher (17%; two of 12 patients) in those patients specifically who had antibodies to bovine thrombin. The number of patients with bleeding events was notably low and the subgroup of patients with antibodies is too small to draw any firm conclusions.

A study of 151 surgery patients exposed to bovine thrombin was conducted by Ortel et al [9], which provided detailed information on the frequency and types of adverse events occurring in surgical patients (109 CABG patients and 42 valve surgery patients) exposed to bovine thrombin. Of the 151 total surgery patients, 7.3% developed excessive postoperative bleeding or had a reoperation for bleeding, 3.3% required transfusion support after postoperative Day 2, and 3.3% experienced gastrointestinal bleeding. When examining the role of risk factors in this patient population, it was determined that prior surgical procedure, increased thrombin exposure during the study, postoperative elevated levels to human coagulation proteins, and postoperative abnormal coagulation study results were not significantly associated with an increased risk of adverse events. While it was determined that having preoperative elevated antibody levels to two or more bovine proteins was a significant risk factor for an incident adverse event, it was conversely also determined that having postoperative elevated antibody levels to two or more bovine proteins was actually associated with a significant decreased risk of adverse events.

There were two randomized trials included in the review that reported complete safety data, including bleeding complications [17,38]. One study compared FloSeal™ to another bovine thrombin product called Gelfoam^® ^(both included thrombin-JMI) in 309 surgery patients who were randomized to the two treatments during surgery [38]. The study did not compare the rates of safety events across the two thrombin-treated study groups, but rather reported the total occurrence of adverse events. In the 309 patients, 2.9% developed excessive post-operative bleeding, 4.9% developed anemia, 0.32% experienced a transfusion reaction, and 0.65% had decreased platelets. The study found no association between the presence or absence of bovine antibodies and the occurrence of bleeding complications at the six- to eight-week follow-up.

The second and most recent randomized trial randomized 206 surgical patients to treatment with bovine thrombin (Thrombin JMI) and 205 patients to treatment with recombinant human thrombin (rhThrombin). At baseline, 5% of the patients randomized to receive bovine thrombin had antibodies to bovine thrombin, while 1.5% of the patients who received rhThrombin had antibodies to human thrombin. At the one-month follow-up, 21.5% of the bovine thrombin group had an antibody elevation; the rhThrombin group had no new antibody elevations. However, there were no significant differences in regards to the incidence of adverse events. In each treatment group, 99.5% of patients reported some adverse event, with 22% of the bovine thrombin and 18% of the human thrombin patients reporting a serious adverse event. There were 11% of bovine-thrombin treated patients that experienced a bleeding event compared to 13% of patients with human thrombin. Rates in this study seemed to be much higher than other studies, but as indicated by 99.5% of patients being noted to have had an adverse event, the safety reporting seemed to be very thorough and may have included a large number of minor adverse events that would not have been noted in the other published surgical studies. It should be noted that the two randomized treatment arms had comparable rates, regardless of the higher numerical values of those rates.

## Discussion

The rates of perioperative bleeding events in studies of surgical patients ranged from 0%–11% in patients definitely exposed to bovine thrombin, with most studies reporting rates between 2%–3%. The rates for the other published studies of surgical patients ranged similarly from 0.1% to just over 20%, with most studies reporting rates between 2%–5%. The risk of bleeds was not clearly different in those studies reporting use of bovine thrombin in all patients compared to the other surgical populations studied. Due to the lack of reporting in the general surgery studies, it should be noted that it is possible that many of the patients in these studies were also exposed to bovine thrombin products.

It was clear that the rates varied with different patient, surgical, and other risk factors. Several studies clearly noted that surgical factors such as the type of surgery [33], complexity of surgery [23], and urgency of surgery [28] affected the risk of bleeding events. Further, patient characteristics such as age, gender, body mass index, comorbid conditions (e.g., cancer, atrial fibrillation), and treatment characteristics such as concomitant medications (e.g., clopidogrel, warfarin, aspirin) are also associated with increased risks of bleeds in surgical patients. The studies that included entire populations of patients treated with bovine thrombin did not appear to demonstrate a higher rate of bleeds than the other published surgical studies, although no formal statistical tests were conducted. Unfortunately, most of the studies examined did not conduct comprehensive analyses of multivariate factors in the examination of the risk of bleeding events in surgery, and thus many of reported bleeding rates may be confounded by a variety of risk factors.

Many of the surgical studies included in our comparator surgical group may have employed the use of bovine thrombin in several of their previous surgeries. Because most studies do not routinely report the use of bovine thrombin in surgery in their publications, there is no way to confirm. Even if these studies did include bovine thrombin patients – and it would be expected that they would because thrombin is such a commonly used product in surgery – if bovine thrombin did increase the risk of bleeding events in surgery, it would be expected that there would be some indication of higher bleeding rates in the bovine thrombin studies; which there is not. This expectation would be to do a comparison of a group of 100% bovine-thrombin treated patients being compared to a group with some unknown proportion exposed to bovine thrombin (but with that proportion clearly being lower than 100%).

We identified all of the published studies within the past 10 years via our comprehensive search methodology that had a clear primary or secondary intention of examining the risk/rate of bleeding events in surgical patients. There are many additional studies that may provide information on the risk of bleeding events in surgical patients as a part of their safety results and conclusions. We did not include these studies in this review because we felt that the only way to ensure that the reporting of bleeding events was complete was to require that the study have this as a clear primary or secondary objective of the study. Without the requirement of focused, detailed reporting, we would have to make assumptions to the completeness of reporting and wouldn't be sure whether the three bleeding events reported in a study were all of the bleeding events, all of the serious bleeding events, or whether the reporting was driven by some other factor that would result in a partial reporting of events.

It should be considered that the definitions of a "bleeding event" differed substantially across studies and that this may lead to difficulty in comparing the rates across studies and treatment group and making scientific conclusions. This is definitely a possibility to explore for more focused comparisons, but even in the studies that report all bleeding events in the surgical population, the rates were still comparable to the bovine thrombin groups, if not lower for bovine thrombin. Prior research has also demonstrated that study design is an important factor in understanding the risk of adverse events, and hence it may be expected that we may see higher rates of adverse events in randomized trials due to active surveillance for the event of interest as compared to retrospective observational studies where the events only present when symptomatic [39]. Although this would still apply here, we believe that this is less of an issue as major bleeding events would be identified regardless of the study design and/or definition of bleeding events.

The evidence in this literature review suggests that the rates of bleeding events in bovine thrombin surgical studies and other published surgical studies are not notably different. Unfortunately, this comparison is far from optimal and the results could be driven by a large number of factors. There are many elements, including definition of bleeding event, type of surgery, patient factors, and other risk factors that can influence the risk of bleeding events in surgical patients. Unless the authors controlled for these factors in their own original research, it is unlikely that they can be compared and evaluated retrospectively from their published work. This indirect comparison of bovine thrombin treated patients to other surgical patients provides suggestive evidence, but is limited in its ability to make definitive conclusions due to the unknown, but expected inter-study variability. None of the bovine thrombin surgical studies provided a comparable non-bovine thrombin treatment group from which to compare, although Chapman et al [17] did compare bovine thrombin to randomized treatment with rhThrombin and found no differences. More prospective studies are needed to accurately estimate the bleeding risks in surgical patients treated with topical bovine thrombin and patients not exposed to topical bovine thrombin, and to establish the risk of bleeding attributed to topical bovine thrombin and to other identifiable factors.

## Abbreviations

The following abbreviations are used throughout this paper: Medical Subject Headings (MeSH), Coronary artery bypass graft (CABG), and Recombinant human thrombin (rhThrombin).

## Competing interests

Matthew Reynolds, Sheila Crean, Srinath Samudrala, and John Clark currently consult for King Pharmaceuticals. This review was funded by an unrestricted research grant by King Pharmaceuticals, Cary, NC 27513

## Authors' contributions

MR, SC, and JC contributed to the conception and design. MR, JC, and SS participated in the acquisition of data. MR, JC, SC, and SS took part in the analysis and interpretation of data. MR and SS drafted the manuscript. SS, SC, and JC provided to critical revision.

## Supplementary Material

Additional file 1 Table 1. Risk of Bleeding Events in Published Surgical Studies (1997–2007). Table 2. Risk of Bleeding Events in Published Surgical Studies of Bovine Thrombin (1997–2007). These tables show the studies examined looking at bleeding events of surgical studies broken down by author, year, surgery type, number of patients, study design, definition of bleeding event, and risk of bleeds Click here for file
